# Overexpression of ginkbilobin-2 homologous domain gene to enhance the tolerance to *Phytophthora cinnamomi* in plants of European chestnut

**DOI:** 10.1186/s12864-025-12485-x

**Published:** 2026-01-09

**Authors:** Susana Serrazina, Mª Teresa Martínez, Silvia Valladares, Lucía Del Castillo-González, Marcelo Francisco, Marta Berrocal-Lobo, Eduardo Piñas, Pablo Piñeiro, Rui Malhó, Rita Lourenço Costa, Elena Corredoira

**Affiliations:** 1 BioISI – Biosystems and Integrative Sciences Institute, Faculty of Sciences of the University of Lisbon, Campo Grande, Lisbon, 1749-016 Portugal; 2 Misión Biológica de Galicia, Consejo Superior de Investigaciones Científicas (MBG-CSIC), Avda Vigo s/n, Campus Vida, Santiago de Compostela, A Coruña, 15705 Spain; 3 Centro para la Biodiversidad y Desarrollo Sostenible (CBDS), ETSI Montes, Forestal y del Medio Natural, Universidad Politécnica de Madrid, Ciudad Universitaria s/n, Madrid, 28040 Spain; 4 Instituto Nacional de Investigação Agrária e Veterinária (INIAV), Quinta do Marquês, Avenida da República, Oeiras, 2780-159 Portugal; 5 Centro de Estudos Florestais, Laboratório Associado Terra, Instituto Superior de Agronomia, Universidade de Lisboa, Tapada da Ajuda, Lisbon, 1349-017 Portugal; 6 Present Address: Agromillora Iberia, Barcelona, Spain

**Keywords:** Agrobacterium tumefaciens, Castanea sativa, Cysteine-rich repeat secretory protein, Genetic transformation, In vitro tolerance assays, Ink disease, Somatic embryos

## Abstract

*Castanea sativa*, a species of high ecological and economic relevance in Europe, faces severe threats from root rot caused by *Phytophthora cinnamomi*. To explore genetic strategies for enhancing disease tolerance, we investigated the functional role of a chestnut gene homologous to *Ginkgo biloba*’s ginkbilobin-2 (*Cast_Gnk2-like*), known for its antifungal properties. Using *Agrobacterium tumefaciens*-mediated transformation, the *Cast_Gnk2-like* gene was introduced into somatic embryos from two embryogenic chestnut lines. Transformation efficiency was genotype-dependent, and varied from 14.2% to 2.5%. Twelve independent transgenic lines were confirmed by PCR, and each was estimated to carry a single copy of the transgene. Gene expression analysis revealed significant *Cast_Gnk2-like* transcript levels in two transgenic lines. Following cold storage and germination treatment, viable transgenic plants were regenerated. Disease tolerance assays demonstrated that *Cast_Gnk2-like* overexpression significantly reduced root necrosis and symptom severity, indicating enhanced tolerance to *P. cinnamomi*. These findings highlight the potential of targeted gene overexpression to improve disease resilience in chestnut through genetic engineering.

**Supplementary Information:**

The online version contains supplementary material available at 10.1186/s12864-025-12485-x.

## Background

Among the chestnut species, *Castanea sativa* Mill. is considered the only native species of the genus in Europe with a circum-Mediterranean distribution that extends from the Atlantic to the Caspian Sea [1]. Although primarily a forest tree, chestnut is also used in agriculture as a cultivated crop. Chestnut trees can appear isolated, forming groves of pure masses or in mixed masses together with different oak species or pines, although it is frequently to appear forming pure masses of anthropic origin called chestnut groves (in Spanish *castañero*s or *soutos* or in Portuguese *castanhais*) [2]. Chestnut agrosystems have been declared of community interest habitat within Directive 92/43/CEE of the European Union [3], to promote their conservation, especially taking into account the threats that weigh on them and their great economic, ecological and social value. The species is valued for its high-quality wood and edible fruits, the chestnuts, whose cultivation is rising due to strong demand and versatile uses (dried, purees, flours, jams, preserves, stews, or marron glacé) [4, 5]. Worldwide production is estimated at 2.21 Mt per year, with contributions from about 46 countries, 27 of them in Europe [6]. In addition, in the last years, chestnut fruits are being used to fed Galician pork from which are yielded high-quality products such as Chestnut-fed Galician ham pork, as well as a variety of premium cold meat [7]. The chestnut tree is also an emblematic species that supports a large number of traditional uses, being part of our culture for centuries as a food resource, essential for people and livestock, as well as an element of construction, crafts and essential fuel in many towns, also constituting one of the best examples of a comprehensive and sustainable productive ecosystem [8]. Chestnut ecosystems are also closely linked with tourism, leisure and well-being, and their exploitation as mentioned before represents a fundamental pillar for rural development in less productive areas, where the creation of employment and generation of income are vital.

Root-rot or ink disease (*Phytophthora spp.*) and chestnut blight (*Cryphonectria parasitica* (Murrill) Barr) are the most significant threats to European chestnut [9]. Due to the economic and ecological importance of this species, breeding programs have long sought to develop *Phytophthora cinnamomi* Rands (Pc)-resistant genotypes [10–14]. Traditional breeding efforts have led to the development of tolerant hybrids through controlled crosses between *C. sativa* and resistant Asian species (*C. crenata* and *C. mollissima*). However, many of these hybrids usually present lower fruit quality compared to pure *C. sativa*. While hybrid rootstocks have proven highly effective in mitigating Pc damage, they have demonstrated limited adaptability to colder and drier conditions in southern areas, where late frosts and droughts can compromise their survival [15]. Despite these limitations, they remain the only viable strategy against ink disease to ensure the sustainability of European chestnut orchards.

One of the main challenges of conventional breeding in trees is the long time required to obtain new tolerant genotypes due to their biological cycle. In European chestnut, the juvenile phase typically lasts 10–15 years, although grafting onto mature rootstocks can substantially reduce the time to flowering and fruiting, often to 3–5 years. However, the overall breeding cycle remains lengthy, as resistance evaluation, selection, and multi-year field validation are still required. To overcome this limitation, modern breeding programs now integrate genomic tools to accelerate the selection process and enhance precision. The use of transcriptomics, genetic mapping, and histopathology has provided key insights into the resistance mechanisms of Asian species, facilitating their transfer to *C. sativa* [16–19]. Notably, transcriptomic analyses identified several defence-related genes, including those involved in pathogen recognition, cell wall reinforcement, hormonal signalling pathways (salicylic acid, ethylene, and jasmonic acid), and antifungal activity. Among them, the *Cast_Gnk2 like* gene of *C. crenata*, encoding an antifungal protein, emerged as a key marker distinguishing resistant from susceptible chestnut genotypes [17]. This gene was subsequently used in *Agrobacterium*-mediated transformation studies of embryogenic chestnut lines, as well as for protein purification and functional validation [20–22].

Genetic transformation of the disease-prone *Castanea* species with antifungal or antimicrobial genes to increase disease resistance or tolerance is a reliable and complementary biotechnological alternative to conventional breeding efforts [23]. In European chestnut a genetic transformation protocol with marker genes was described for the first time by [24], and later optimized by [25, 26] through somatic embryogenesis. Subsequently, since the genes involved on chestnut tolerance to Pc and *C. parasitica* were unknown, two pathogenesis-related proteins, namely a thaumatin-like protein [27] and a chitinase [28], were overexpressed in chestnut somatic embryos to assess their impact on the diseases affecting this species. However, in the last few years, considerable information about the mechanisms and genes involved on tolerance to Pc has been described, as the already focused *Cast_Gnk2-like*, that may play a role in resistance to this oomycete pathogen. *Cast_Gnk2-like* encodes a cysteine-rich repeat secretory protein and notably shares homology with Ginkbilobin2 (Gnk2), produced in gymnosperms in response to both biotic and abiotic stresses [29], further underscoring its potential involvement in stress-related defence mechanisms in other plant species. In *Ginkgo biloba*, Gnk2 is a secreted protein which binds with high affinity to D-mannose, being related with the attachment and disturbance to the cell wall of plant pathogenic fungi [29]. In previous reports, our group has overexpressed this antifungal gene on somatic embryos of holm oak and cork oak, and plants that overexpressed *Cast_Gnk2-like* survived more days than wild type controls during in vitro tests with the pathogen [20, 22].

After the functional analysis studies of *Cast_Gnk2-like* in Pc-susceptible oaks, it’s now the turn of *C. sativa*, with the objectives of the present work being the following: 1) Genetic transformation of *C. sativa* embryogenic cultures with the antifungal gene *Cast_Gnk2-like* and evaluation of transformation efficiency; 2) Molecular characterisation of transgenic lines; and 3) Plant regeneration and evaluation of their tolerance to Pc.

## Materials and methods

### Plant material

Embryonic lines named CI-3 and CI-9 were used as a source of explants to carry out the transformation experiments. These lines were initiated from distinct zygotic embryos, as was described in [23]. They were maintained by secondary embryogenesis as described in [23] with subcultures at 6-week intervals in proliferation medium defined as Murashige and Skoog medium [30] with half-strength macronutrients and supplemented with 0.1 mg/l benzyladenine (BA), 0.1 mg/l naphthaleneacetic acid (NAA), 3% sucrose (w/v), 0.7% Agar Sigma (w/v; A-1296, Sigma-Aldrich, St. Louis, MO, USA). The embryogenic lines were cultured under a 16-h photoperiod (provided by cool-white fluorescent lamps at a photon flux density of 50–60 µmol m^− 2^ s^− 1^) and 25 °C light/20°C dark temperatures (standard conditions).

### *Phytophtora cinnamomi* culture

*Phytophtora cinnamomi* strain was kindly provided by TRAGSA (Maceda nursery, Orense, Spain) and isolated by the Center for Research and Technology of Extremadura (CYCYTEX), located in Mérida, Spain. The *P. cinnamomi* mycelium culture was obtained from a fresh plate using previous describe methodology [31–33]. Zoospores were generated following our optimized protocol, as detailed at [34]. Briefly, the *P. cinnamomi* mycelium was cultured on potato dextrose agar in the dark at 24 °C for 3 weeks. The mycelium was then transferred to a V8 agar medium and incubated in the dark at 24 °C for 7 days on a Miracloth disc. To induce sporangia formation, the Miracloth disc with the mycelium was moved to a V8 clarified liquid medium under fluorescent light with shaking at 24 °C for 48 h. This was followed by a second sporangia induction step in mineral salt solution enriched with chelated iron, under fluorescent light and shaking at 24 °C for 24 h. For zoospore induction, the Miracloth disc containing *P. cinnamomi* sporangia was transferred to sterile water and subjected to shaking at 4 °C for 90 min. Fresh zoospores were then collected by filtration. The zoospore stock was obtained, and stored at − 80 °C until use, following the previous optimized protocol at [34].

### Plasmid construction and *Agrobacterium tumefaciens* strain

The Gateway system (Invitrogen, USA) was used to clone the *Cast_Gnk2-like* coding sequence into the plasmid pK7WG2D [35] under the CaMV35S promoter, as described by [23]. The vector also includes the neomycin phosphotransferase (*NPTII*) selective gene, and the green fluorescent protein (*EGFP*) reporter gene, used for the selection of transformed embryos. The final vector, denominated as pK7WG2D-GIN (see Supplementary information 1), was transferred into *Agrobacterium tumefaciens* strain EHA105 [36] by the freeze-thaw method [37] and used in the transformation experiments.

### Transformation of somatic embryos

For transformation, the procedure described in [23] was applied, starting from explants that consisted of small clumps of 2 or 3 somatic embryos in globular or early-torpedo stage, isolated from two chestnut embryogenic lines. *Agrobacterium* strain and infection culture was prepared following the procedure described by [23]. For each embryogenic line, 120 explants were used (6 Petri dishes with 10 explants and the experiment was repeated twice) resulting in a total of 240 explants. Additionally, for each line 20 explants that were not infected and were cultured on proliferation medium without antibiotics (positive control) and with antibiotics (negative control). Explants were co-cultured for 5 days in darkness with the *Agrobacterium tumefaciens* strain EHA105pK7WG2D-GIN. Then, bacteria were eliminated by a wash with sterilized water supplemented with 300 mg/l cefotaxime for 30 min, and subsequently explants were transferred to selective proliferation medium with 100 mg/l kanamycin (Kan), 200 mg/l cefotaxime and 300 mg/l carbenicillin. Cultures were subcultured every two weeks for a total duration of 10 weeks. Kan-resistant explants were subsequently identified, and the percentage of Kan-resistant explants (defined as the proportion of initial explants exhibiting Kan tolerance) was calculated.

### Selection and establishment of transgenic lines

Kan-resistant explants were isolated and sub-cultured on selective medium with 150 mg/l Kan to increase the selective pressure. The transformation efficiency (TE, defined as the percentage of initial explants that showed fluorescence [GFP+]) were determined after 14 weeks. Fluorescence was observed using a Leica M205 FA magnifier (Germany) equipped with a 200 W lamp and a specific fluorescence filter, with an excitation of 470/40x nm and an emission of 525/50 nm. Images were obtained with a Leica DSC7000T camera (Germany). A single somatic embryo at the cotyledonary stage was isolated from each GFP + explant. Each embryo was proliferated by secondary embryogenesis in selective medium to establish the different transgenic embryogenic lines. Twelve weeks after isolation Kan was removed and transgenic lines were multiplied on proliferation medium.

### Molecular analysis of transformed embryogenic lines

#### Gene presence analysis

The presence of the transgenes (*Cast_Gnk2-like* gene and marker genes *GFP* and *NPTII*) was confirmed by PCR amplification, as described in [20]. DNA was obtained from transformed and untransformed (WT) somatic embryos using the Qiagen DNeasy Plant Mini Kit (Qiagen, Germany). Once extracted, the DNA concentration was quantified using a Nanodrop ND-2000 spectrophotometer (Thermo Fisher Scientific, USA). All amplification reactions were carried out in an MJ Mini thermal cycler (BioRad, USA), using a 25 µl reaction volume. The reaction consisted of 250–500 ng of genomic DNA, 0.5 mM of MgCl, 1U of Taq DNA Polymerase (Qiagen, Germany), 2.5 mM of dNTPs, and 15 µM of primers. The primer sequences, the amplification programs used, the fragment size generated are described in Supplementary information 2. The PCR amplification products were resolved in 1.5% (w/v) agarose gels.

#### Gene copy number estimation

The number of *Cast_Gnk2-like* copies inserted into embryogenic transformed lines was estimated using quantitative real-time PCR (qPCR), as described by [20]. DNA was extracted as outlined in the previous section, and three biological replicates per line were prepared. Specific oligos targeting *NPTII* were utilized in the reactions (see Supplementary Information 2). A standard curve was established following the method of [38], by mixing the plasmid used for transformation with non-transformed genomic DNA. Based on the genome size of *C. sativa* reported by [39], we calculated the amount of plasmid required to be mixed with non-transgenic DNA to simulate 1, 2, 5, and 10 copies of the transgene. The qPCR cycling included a subsequent step for melting curve analysis. The qPCR experiments incorporated no template controls, two technical replicates, and two repetitions. The copy number in each sample was estimated by making the correspondence between the C_T_ values and the simulated copies of the transgene.

#### Gene expression analysis

The expression of *Cast_Gnk2-like* was analyzed using qPCR. Total RNA was extracted from non-transformed and transformed lines, utilizing somatic embryos at the early cotyledonary stage. The extraction followed a CTAB buffer protocol as outlined by [22]. Three biological replicates were taken from three distinct embryo clumps. The extracted RNA was treated with the TURBO DNA-free™ Kit (Invitrogen, USA) to remove any potential DNA contamination. RNA integrity was confirmed on 1% (w/v) agarose gels. Total RNA concentration was measured using a NanoDrop™ One Microvolume UV–Vis Spectrophotometer (Thermo Fisher Scientific, USA). Three µg of total RNA were used as the template for reverse transcription with RevertAid H Minus Reverse Transcriptase (Thermo Fisher Scientific, USA), primed with an oligo(dT)_23_. Specific primers for *Cast_Gnk2-like* are detailed in the Supplementary Information 2. For each reaction, 1.2 ng of cDNA were used in a 15 µL final volume, which included 7.5 µL of NZYSupreme qPCR Green Master Mix (2x) (NZYtech, Portugal). Each primer was used at a final concentration of 0.2 µM in a CFX96 Touch Real-Time PCR Detection System (BioRad, USA). The qPCR reactions began with a denaturation step at 95 °C for 10 min, followed by 40 cycles of denaturation at 95 °C for 15 s and annealing for 30 s. Each set of reactions included a no-template control, two technical replicates and two repetitions. Dissociation curves were used to check for non-specific PCR products. To normalize the expression data, *Elongation factor 1-alpha* (*EF1α*) and *Actin* (D. Matthews, personal communication, August 24, 2023) were used as reference genes (Supplementary Information 2). Gene expression was calculated using the Hellemans method [40].

### Plant regeneration of transgenic embryos

Germination and plant regeneration was performed following the protocols defined by [41]. Cotyledonary embryos (>5 mm) were isolated and matured on maturation medium for further development. After 4 weeks, they were cold-stored at 4 °C in dim light conditions (8 µmol m⁻² s⁻¹) for 2 months, before being cultured on germination medium. Plant conversion rates were generally low, but some embryos developed shoots. These shoots were multiplied via axillary budding proliferation and rooted following [23]. Briefly, elongated shoots were rooted in rooting medium defined as GD medium (Gresshoff and Doy, 1972; Duchefa, Netherlands) [42] with third-strength macronutrients supplemented with 25 mg/l IBA and 0.6% BD Agar Difco (w/v; Difco Laboratories, New Jersey, USA). Shoots were cultured in rooting medium for 48 h in darkness and later transferred to auxin-free rooting medium. Fluorescence was also tested on leaves from transgenic and non-transgenic plants (control) as described before for somatic embryos.

### In vitro tolerance assays

#### Plant growth conditions

Plants obtained as described above were transferred from agar to plastic sterile tubes containing 1 ml of sterilized water and they were incubated in an Aralab chamber (Lisbon, Portugal), at 50% humidity, temperature of 24 °C during the day and 18 °C during the night, with a 16-h light/8-h dark photoperiod and light intensity of 150 µmol m^− 2^ s^− 1^ for all experiments. They were maintained in these conditions for a week for acclimation.

#### Plant infection

Plants were transferred to sterile DeWit^®^ culture tubes (Sgl, Barcelona, Spain), containing 1 ml of sterile distilled water and maintained for an additional three days for further acclimation. The plants were divided into two groups: a control group and an inoculated group. The inoculated plants were transferred to culture tubes with 1 ml of Pc zoospores at a final concentration of 10^7^ zoospores/ml, while control plants were transferred to sterilized water, following a previous optimized protocol [34].

#### Disease evaluation symptoms

The plant disease symptoms produced by Pc were considered following the standards approved for diagnostic protocols for regulated pests by EPPO council [43]. Those symptoms included principally root rot brown lesions followed by necrosis produced directly by the pathogen and secondary symptoms of decline producing leave chlorosis, high necrosis and death. Disease symptoms were followed for 7 days using a modified infection criteria described by [44] as followed: 0. No symptoms, 1. Leaf chlorosis and light necrosis on roots, 2. Apparent necrosis on leaves and light necrosis on roots, 3. High necrosis on leaves and roots, 4. Decomposed plants.

Fresh weight (FW) was measured at 7 dpi, to calculate the fresh weight lost (FWL) Following the formula:

$$\begin{aligned} FW\left(\%\right)=&\frac{\left(FW\;7dpi\;\times100\right)}{FW\left(T0\right)};\\&\;FWL\left(\%\right)=100\%-FW\left(\%\right) \end{aligned}$$

Photographs of the explants were captured for assessing root necrosis (RN). The quantification of root infection and necrosis involved measuring the total length of the infected root and the segments showing necrosis at 7 dpi using the software ImageJ. The calculations were performed using the following formula:

$$\begin{aligned} &Root\;necrosis\left(\%\right)= \frac{\left(Length\;of\;infected\;area\;\left(cm\right)\;\times\;100\right)}{Total\;length\;\left(cm\right)} \end{aligned}$$

Necrotic lesions were measured by Trypan blue staining, performed as previously described [45]. Roots were stained with Trypan Blue solution for 20 min, rinsed with 100% ethanol over two days, and preserved in 60% glycerol until microscopic observation. At least six photos were quantified per root area and treatment. Image processing was performed using ImageJ Software and specific plug-in tools for measuring root necrosis as previously described in [46].

### Statistical analysis

**Fig. 1:**
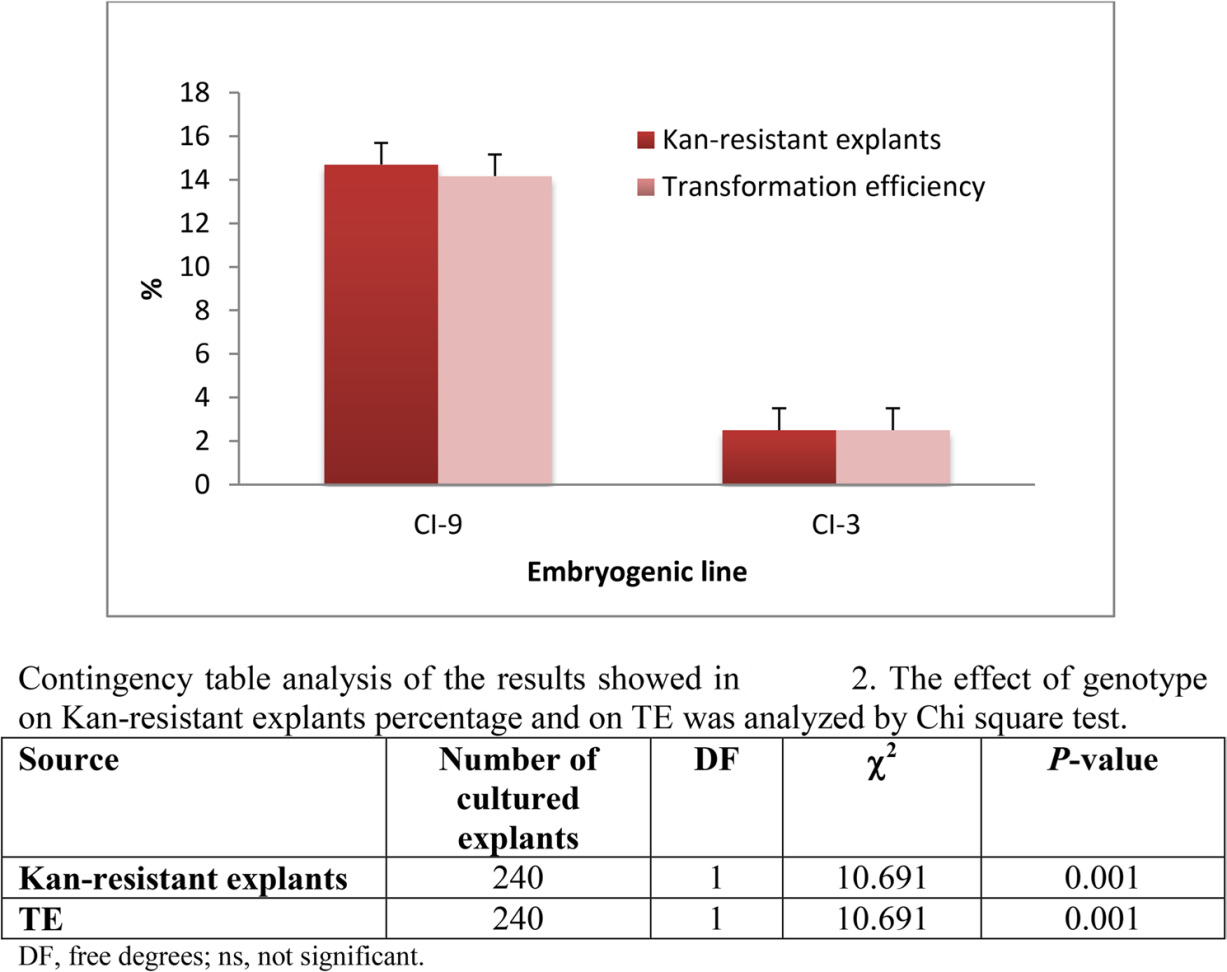
Percentage of Kan-resistant explants and transformation efficiency (TE) of the 2 embryogenic chestnut lines obtained following cocultivation for 5 days with the strain EHA105 pK7WG2D-GIN. In each line, columns represent means ± SE of 120 explants. Kan-resistant percentage was determined after 10 weeks, and the TE was evaluated after 14 weeks. The statistical analysis of these data is showed in the table

The effect of genotype on genetic transformation data was analyzed using G-test (Fig. 1). Gene expression levels were analyzed by Kruskal-Wallis test, followed by a Dunn’s test for significance analysis of the transgenic lines compared to the untransformed genotypes at *p*-value < 0.05 (Fig. 4). Both analyses were performed using the SPSS 29.0 statistical package for Windows (IBM, Armonk, NY, USA). In the tolerance assay, the Stat Graphics Centurion XVI.II program (Stat Point Technologies, Inc., Warrenton, VA, United States) was used for all data analysis related to plant fresh weight lost and root necrosis. ANOVA I and Duncan’s mean comparison test were performed for all experiments and t-tests with a significance level of 0.05% (Figs. 5 and 6). In ANOVA analyses, percentages were subjected to square-root transformation prior to analysis to normalize the data. In the case of non-homogeneous variance, a non-parametric Kruskal–Wallis test was used.

## Results

### Genetic transformation of somatic embryos

**Fig. 2:**
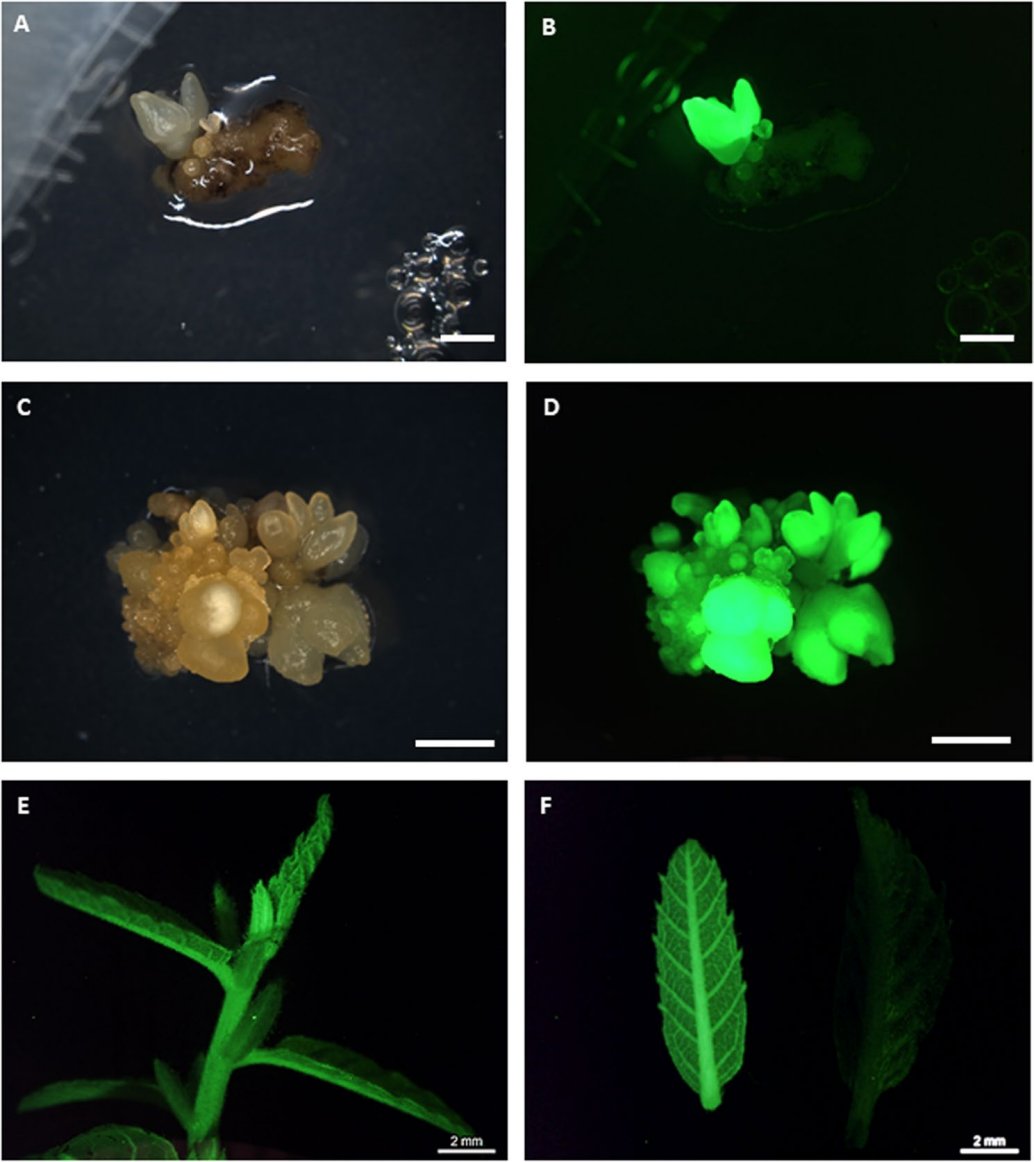
Genetic transformation of chestnut somatic embryos with *Cast_Gnk2-like* gene. **A**, **C**: Kan-resistant somatic embryos obtained from CI-3 (**A**) and CI-9 (**C**) lines after 14 weeks on selective medium with kanamycin. **B**, **D**: The same transgenic somatic embryos of line CI-3 (**B**) and CI-9 (**D**) showing GFP fluorescence. **E**: GFP expression on an apex of transgenic plant. **F**: GFP expression on a leaf of transgenic plant (left) and untransformed plant (right). **B**, **D**, **E**, **F**: visualization on an epi-fluorescence stereomicroscope under UV light. **A**-**D**: Bar 2 mm

Kan-resistant somatic embryos were detected after 10 weeks in both lines (Fig. 1), but the two lines exhibited distinct behaviours during selection. After initiating culture in the selection medium, embryo explants turned brownish/blackish, particularly in line CI-3. New growth was observed in CI-9 line within 4 weeks, with the number of Kan-resistant explants increasing over time. In contrast, CI-3 required at least 8 weeks to show new growth. After 10 weeks, Kan-resistant frequency was determined and it was found to be significantly influenced (*p* ≤ 0.001) by genotype of embryogenic lines (Fig. 1). As result of this, line CI-9 exhibited frequencies of 14.2%, whereas line CI-3 showed significantly lower values of 2.5%. Kan-resistant explants were isolated from both lines and subcultured for an additional four weeks on selective medium to promote embryogenic development and confirm kanamycin tolerance (Fig. 2A, C).

The transformation efficiency assessed based on the fluorescence of surviving explants (Fig. 2B, D) was also genotype-dependent (Fig. 1). The TE was significantly higher (*p* ≤ 0.001) in CI-9 line (14.2%) than in the CI-3 line (2.5%) (Fig. 1). A total of 12 independent transformed lines were established. Successful embryo proliferation by secondary embryogenesis occurred in all established transgenic lines. After 6 weeks on selective medium, the number of secondary somatic embryos per explant was recorded to assess multiplication capacity (data not shown) and embryogenic lines with the highest multiplication ability were selected for further research: two transgenic lines from genotype CI-3 (CI-3-G1 and CI-3-G3) and four transgenic lines from genotype CI-9 (CI-9-G1, CI-9-G4, CI-9-G11, and CI-9-G12).

### Molecular analysis of transgenic lines

**Fig. 3:**
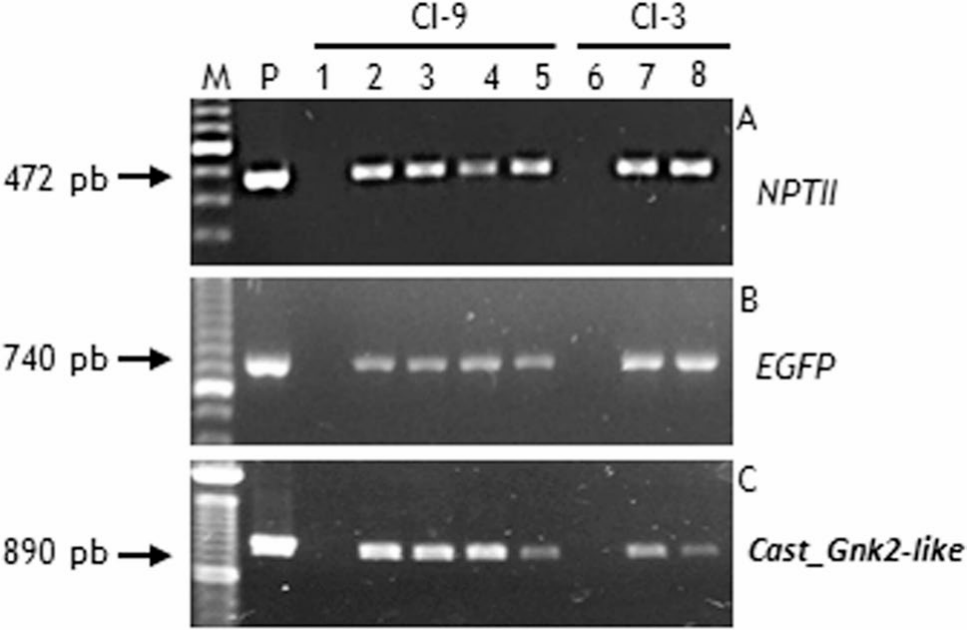
PCR-based detection of the presence of transgenes in putative chestnut transgenic lines. **A**: Amplification of NPTII product of 472-bp. **B**: Amplification of EGFP product of 740-bp. **C**: Amplification of Cast_Gnk2-like product of 890-bp. M: DNA size marker; Lanes 1, 6: DNA from non-transgenic somatic embryos (negative control). P: DNA from plasmid (positive control). Lanes 2, 3, 4, 5, 7, 8: DNA from embryogenic transgenic lines

The presence of transgenes (*Cast_Gnk2-like* gene and marker genes *GFP* and *NPTII*) in genomic DNA of transgenic embryogenic lines with high proliferation ability was confirmed by PCR (Fig. 3). PCR amplification was obtained in all chestnut transgenic lines tested, as well as in the plasmid (positive control), but not in the untransformed lines (negative control) (Fig. 3). The estimation of *Cast_Gnk2-like* copy number in embryogenic transformed lines was obtained through the amplification of an *NPTII* fragment, which is adjacent to *Cast_Gnk2-like* in the T-DNA (Supplementary information 1). All the transformed lines have the same number of transgenes in their genome, estimated as one copy (Table 1). As expected, non-transformed genotypes have zero copies of *NPTII* gene.

**Table 1:** Estimated copy number in *C. sativa* somatic embryogenic lines transformed with *Cast_Gnk2-like* gene

Line	C_T_ Mean	C_T_ Standard deviation	Estimated copy number^1^
CI-3-WT	32,71	2,32	0
CI-3-G1	23,95	1,03	1
CI-3-G3	23,37	0,48	1
CI-9-WT	30,22	0,51	0
CI-9-G1	22,35	0,64	1
CI-9-G4	22,47	0,46	1
CI-9-G11	23,65	0,73	1
CI-9-G12	23,74	1,40	1

^1^values from the correlation between the quantity of plasmid and the number of copies in *C. sativa*, as described in material and methods

**Fig. 4:**
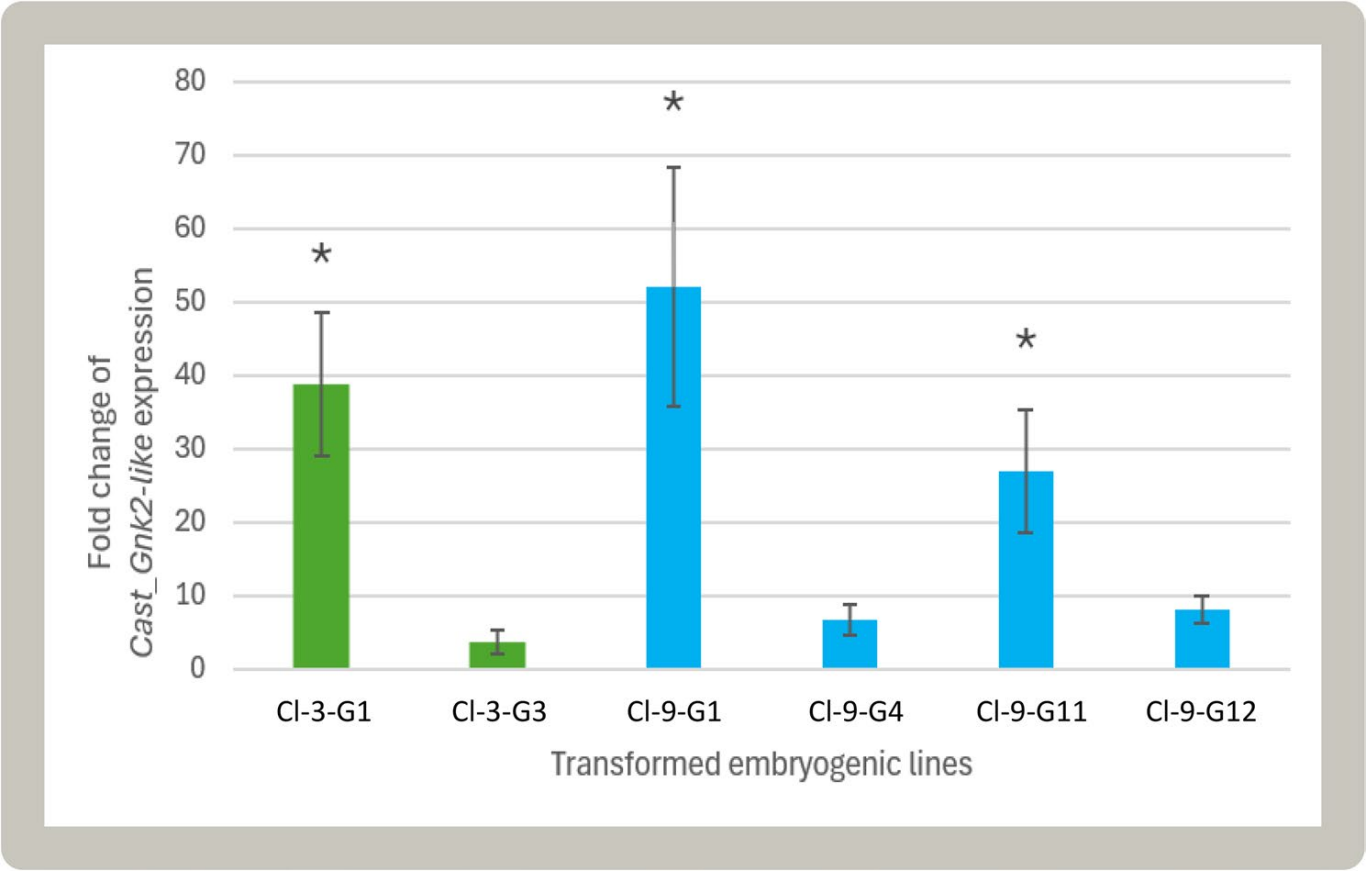
Fold change of Cast_Gnk2-like expression among transformed lines, relative to the respective non-transgenic lines. Green: transformed lines from the CI-3-WT genotype; Blue: transformed lines from the CI-9-WT genotype. Asterisk indicates that the *p*-value obtained from the Kruskal-Wallis post hoc test is lower than 0.05 and hence considered statistically significant

Transgene expression in each transformed line was compared to the corresponding non-transformed genotypes in somatic embryos (Fig. 4). *Cast_Gnk2-like* oligos used in qPCR were designed from the coding sequence of *C. crenata*. Since *C. sativa* and *C. crenata* belong to the same genus, both endogenous and transgene transcripts may be amplified in the same reaction. However, *Cast_Gnk2-like* had CaMV35S as the promoter, which has a constitutive action, so an expression level in transformed lines higher than in non-transformed genotypes was expected. This was true and significant for the CI-3-G1, CI-9-G1, and CI-9-G11 transformed lines (Fig. 4), indicating that these lines exhibit overexpression of the putative resistance gene *Cast_Gnk2-like*.

### Plant regeneration from transgenic somatic embryos

Regeneration occurred in all transgenic lines after two months of cold storage and subsequent transfer to germination medium, except CI-9-G4 (Table 2). Total regeneration (shoot and plant conversion) was higher in the non-transformed lines than in the transformed lines (Table 2). The frequency of plant regeneration was low across all transformed lines and in CI-9-WT, with percentages ranging from 4.2% to 16.6%. Only an acceptable plant regeneration percentage was observed in line CI-3-WT (37.5%). In all cases, regenerated shoots were multiplied via axillary shoot proliferation, enabling unlimited production of transgenic shoots for rooting and acclimatization. GFP expression was also detected in shoots and leaves isolated from transgenic plants (Fig. 2E, F). As expected, no green fluorescence was observed in leaves isolated from non-transformed regenerated plants (Fig. 2F).

**Table 2:** Plant regeneration performance with only root development, only shoot development and conversion into plantlets (root and shoot development) of different chestnut transformed and untransformed lines (WT) after 8 weeks on germination medium

LINE	ONLY ROOT		ONLY SHOOT		CONVERSION (root + shoot)	TOTAL PLANT REGENERATION¹
	(%)	RL (mm)	(%)	SL (mm)	(%)	%
LINES CI-3						
CI-3-WT	33.3 ± 6.5	14.7 ± 3.6	12.5 ± 5.7	7.5 ± 0.3	25.0 ± 11.3	37.5
CI-3-G1	20.8 ± 3.3	12.2 ± 1.6	8.3 ± 3.3	6.5 ± 0.8	4.2 ± 3.3	12.5
CI-3-G3	25.0 ± 5.7	10.8 ± 1.5	4.2 ± 3.3	7.0 ± 0.6	4.2 ± 3.3	8.4
LINES CI-9						
CI-9-WT	20.8 ± 3.3	6.8 ± 0.4	8.3 ± 3.3	7.5 ± 0.6	8.3 ± 0.6	16.6
CI-9-G1	8.3 ± 3.3	7.5 ± 0.8	4.2 ± 3.3	7.0 ± 0.0	0.0 ± 0.0	4.2
CI-9-G4	8.3 ± 3.3	8.0 ± 0.0	0.0 ± 0.0	-	0.0 ± 0.0	0.0
CI-9-G11	12.5 ± 5.7	7.8 ± 0.4	4.2 ± 3.3	8.0 ± 0.0	0.0 ± 0.0	4.2
CI-9-G12	16.7 ± 3.3	7.3 ± 0.9	4.2 ± 3.3	7.5 ± 0.0	4.2 ± 3.3	8.4

Each value represents the mean ± standard error of 3 replications with 8 somatic embryos in each replicate

*RL* root length, *SL* shoot length

^1^Total Plan Regeneration is obtained from the sum of only shoot rate and plant conversion rate

### Disease ratio analysis of transformed plants

**Fig. 5:**
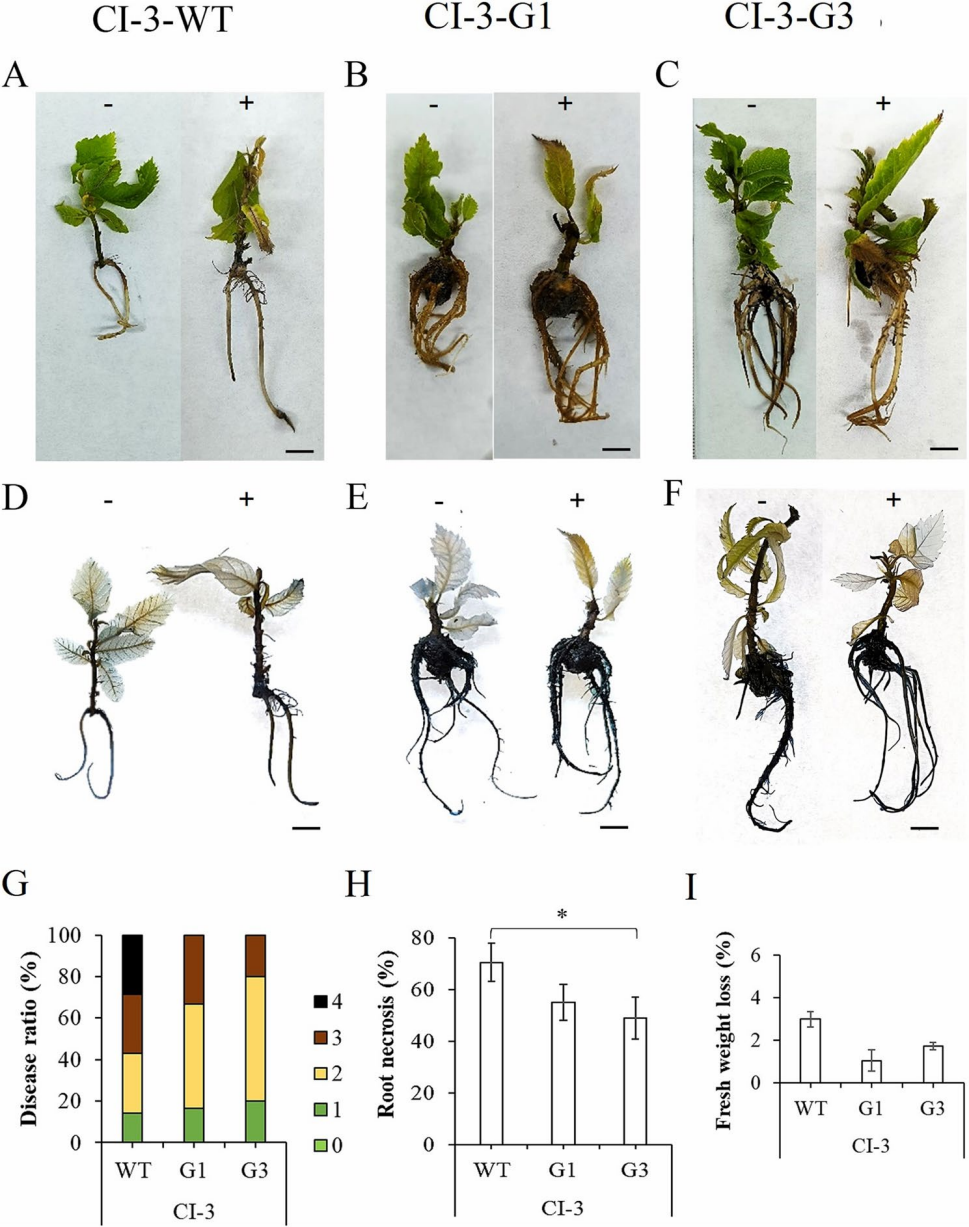
Evaluation of *Phytophthora cinnamomi* tolerance in transgenic plants derived from the CI-9 genotype. Plants of CI-9-G1, CI-9-G11 and CI-9-WT were infected with Pc zoospores (10⁷ zoospores/mL) to assess their level of tolerance to the pathogen. **A**-**C**. Symptoms on explants after 7 days post-inoculation (7 dpi). Bars: (1 cm). **D**-**F**. Detail of cell death symptoms on explants stained with Trypan Blue (TB). Bars: (1 cm). **G**. Disease symptoms expressed in percent related to the total number of explants per treatment (*n* = 6 per each genotype and each treatment), at 7 dpi, where: 0. No symptoms, 1. Leaf chlorosis and light necrosis on roots, 2. Apparent necrosis on leaves and light necrosis on roots, 3. High necrosis on leaves and roots, 4. Decayed explant. **H**. Root necrosis percentage on explants at 7 dpi. **I**. Fresh weight lost expressed in percentage related to controls at 7 dpi. The * in H denotes a statistically significant difference using variance check (*P*-value ≤ 0.05) and Tukey HSD test

**Fig. 6:**
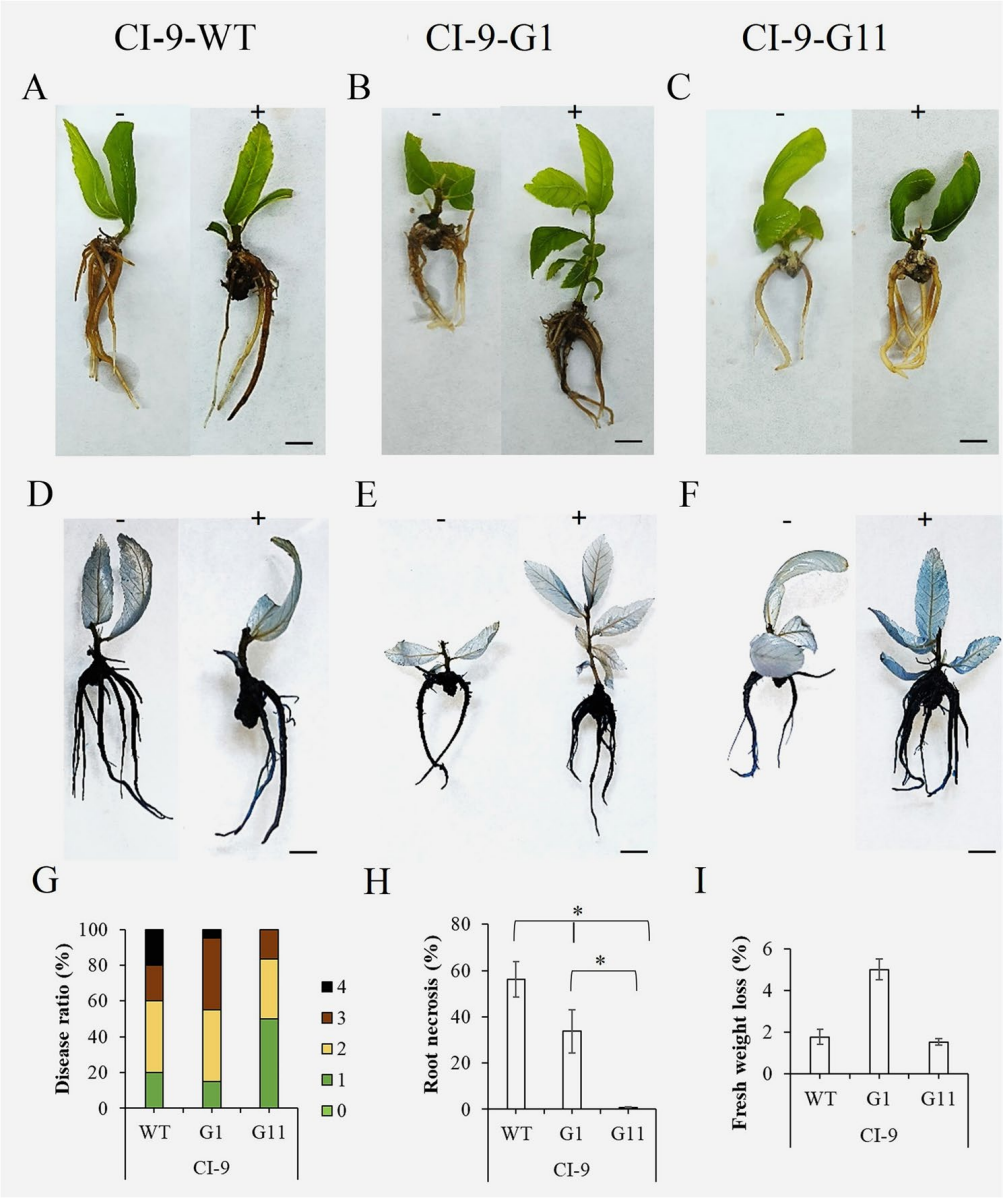
Evaluation of Phytophthora cinnamomi tolerance in transgenic plants derived from the CI-9 genotype. Plants of CI-9-G1, CI-9-G11 and CI-9-WT were infected with Pc zoospores (10⁷ zoospores/mL) to assess their level of tolerance to the pathogen. **A**-**C**. Symptoms on explants after 7 days post-inoculation (7 dpi). Bars: (1 cm). **D**-**F**. Detail of cell death symptoms on explants stained with Trypan Blue (TB). Bars: (1 cm). **G**. Disease symptoms expressed in percent related to the total number of explants per treatment (*n* = 6 per each genotype and each treatment), at 7 dpi, where: 0. No symptoms, 1. Leaf chlorosis and light necrosis on roots, 2. Apparent necrosis on leaves and light necrosis on roots, 3. High necrosis on leaves and roots, 4. Decayed explant. **H**. Root necrosis percentage on explants at 7 dpi. **I**. Fresh weight lost expressed in percentage related to controls at 7 dpi. The * in H denotes a statistically significant difference using variance check (*P*-value ≤ 0.05) and Tukey HSD test

The analysis of the overexpressing lines CI-3-G1, Cl-3-G3, CI-9-G1 and Cl-9-G11, transformed with *Cast_Gnk2-like* gene under CaMV35S promoter control, produced lines with no apparent differences from non-transformed plants n terms of plant growth and development, allowing the subsequent analysis of resistance to Pc. The analysis revealed less statistically significant root necrosis caused by Pc in transformed lines when compared to the untransformed controls, although differences into levels of disease symptoms were observed (Figs. 5 and 6). Differences in the progression of symptoms among CI-3-WT, CI-3-G1, and CI-3-G3 lines, such as chlorosis and necrosis, are visible in Fig. 5A–C. The root necrosis induced by the oomycete was evident in CI-3-WT lines and was confirmed by the trypan blue staining of root death cells, being lower in transformed plants (Fig. 5D–F). The symptoms observed in roots were quantified by disease ratio, which is shown at Fig. 5G. This data reveals a significantly lower disease severity and root necrosis than CI-3-WT (Fig. 5H). Although transformed lines Cl-3-G1 and Cl-3-G3 exhibited an apparently lower fresh weight loss compared to the WT, the differences were not statistically significant (Fig. 5I). Considering all the data, CI-3-G3 showed higher tolerance than G1, although the differences were not statistically significant.

In Fig. 6 a similar analysis of the transformed lines CI-9-G1 and CI-9-G11, with the background Cl-9-WT, is shown. The results illustrate that the symptoms in both *Cast_Gnk2-like* overexpressing lines in response to Pc after 7 days were less severe relatively to the WT plants. Actually, lines CI-9-G1 and CI-9-G11 showed less root necrosis and lower disease indexes (Fig. 6G-H), especially line CI-9-G11 (Fig. 6A-C). Those results were in line with the levels of cell death on roots confirmed by trypan blue staining (Fig. 6D-F). Line CI9-G1 showed much less differences with the WT, however still significant (Fig. 6H). The differences in fresh weight loss between line CI-9-G11 and the WT were not statistically significant. By contrast, line CI-9-G1 presented a higher fresh weight loss compared to the WT, suggesting a possible pleiotropic effect in this line produced by the insertion (Fig. 6I).

Overall, CI-3-G3 and CI-9-G11 consistently demonstrated reductions in root necrosis upon Pc infection compared with WT, pointing that overexpression of the *Gnk2* homolog (*Cast_Gnk2*-like) has a positive effect in the defense to the pathogen. Variability in resistance among the different lines indicates that further molecular analyses are needed to fully understand the observed differences.

## Discussion

Pathogens as fungi, oomycetes, bacteria, and viruses compromise plant health and survival leading to significant economic losses [47]. Among these, ink disease, primarily caused by the oomycete Pc, has severely affected chestnut populations for many years. Since chemical treatments with fungicides and pesticides have proven largely ineffective, developing genotypes tolerant to the pathogen represents the most promising strategy. Biotechnological tools such as somatic embryogenesis and genetic transformation provide effective approaches for developing disease-tolerant genotypes especially in woody plants and for gaining insight into the mechanisms by which plants defend themselves against pathogens [48]. The overexpression of antifungal proteins represents an interesting strategy to enhance plant tolerance against fungal diseases, with potential applications in agricultural biotechnology [49].

In this study, the overexpression of the *Cast_Gnk2-like*, homologous to an antifungal protein, was obtained in the two embryogenic lines tested, although the transformation rates were influenced by the genotype. The effect of genotype on transformation frequencies has also been reported in other woody species, such as American chestnut [50] and pedunculate oak [51], emphasizing the importance of genetic background in determining the success of genetic transformation procedures. The transformation rates in chestnut were higher than those previously mentioned in holm oak [20] and cork oak [22] using the same construct and the same *Agrobacterium* strain. The use of the GFP as a marker gene enabled direct visual selection of transformed embryos, facilitating their identification. Although GFP has been reported to exhibit cytotoxic effects in some plant cells [52], evidence obtained in the present paper and other species like pear [53], rubber tree [54], holm oak [55], and cork oak [56] indicate that GFP does not negatively affect embryo development or plant regeneration.

The conversion of somatic embryos into plants remains a major limiting factor in most embryogenic systems of woody species [57]. This limitation is even more pronounced in transgenic somatic embryos [27, 58, 59], likely due to extended culture in kanamycin-containing media [60]. Although conversion frequencies in European chestnut were low, the resulting shoots could be excised and easily multiplied through axillary bud proliferation and were subsequently rooted, thus producing viable plants for tolerance assays. A similar approach was also successfully used in cork oak for the regeneration of plants from somatic embryos overexpressing the *CsTL1* gene, which encodes a thaumatin-like protein [56].

The number of *Cast_Gnk2-like* gene copies in transgenic lines was estimated to be one. Low copies of the chestnut thaumatin-like protein gene (one and two) were also obtained with *C. sativa* somatic embryos in our previous study [27]. One copy of the inserted gene is important to avoid transgene silencing. Despite the single number of *Cast_Gnk2-like* copies, the expression level was notably different among transgenic lines. Besides copy number, other factors may influence transgene expression, such as the position and integration of the T-DNA in the genome, and the use of the CaMV35S promoter [61]. The promoter was used to confer the overexpression of *Cast_Gnk2-like* gene, which was essential to proceed with the gene functional analysis. Expression levels of overexpressed genes considerably differ among different transgenic lines, even when the same gene and promoter are used. Therefore, it is essential to evaluate multiple lines to select those with adequate expression levels, as only lines surpassing a minimum threshold typically exhibit the desired phenotype, such as enhanced pathogen resistance.

The tolerance of the lines overexpressing the *Cast_Gnk2-like* gene was evaluated in regenerated plants through an in vitro assay. In vitro testing techniques represent a valuable tool for the identification and selection of cultivars with tolerance or resistance to biotic and abiotic stresses [62, 63]. This approach offers significant advantages over field trials, as it allows for faster, more cost-effective evaluations under controlled conditions, thereby reducing the time, space, and resources required, as well as the avoidance of pathogen escape. Moreover, the controlled laboratory environment enables the uniform reproduction of specific stress conditions, improving both the accuracy and reproducibility of the results. In the present study, in vitro assays were performed using previously isolated stocks of virulent zoospores of Pc, using an optimized protocol [34], enabling a more precise and reliable assessment of plant tolerance compared to previous methods that relied on mycelium plugs. The in vitro tests revealed that the insertions introduced into the transformed plants with *Cast_Gnk2-like* gene, improved the tolerance to Pc when compared to the non-transformed. However, the lines CI-3-G3 and CI-9_G11 were significantly more tolerant than CI-3-G1 and CI-9-G1. These less tolerant lines exhibited *Cast_Gnk2-like* fold change over 30-fold higher, whereas the more tolerant showed lower overexpression. This suggests that Cast_Gnk2-like may induce trade-off during the progression of infection, potentially compromising the efficiency of the plant’s immuneresponses. In some cases, such as in line CI-9-G1, the transformation may have triggered pleiotropic effects that altered plant development. For example, the lower fresh weight loss observed in this line during infection could be a consequence of a slight increase in shoot growth. Rapid shoot growth or accelerated flowering shortly after pathogen exposure is often observed in plants attempting to survive stress. Further molecular analysis of the transgene insertion is therefore required to confirm whether these developmental changes are linked to the observed variation in tolerance.

In this study, the enhanced tolerance to Pc observed in lines CI-3-G3 and CI-9-G11 confirms the relevance of *Cast_Gnk2*-like gene overexpression as part of a strategy for plant protection against the pathogen. Similarly, overexpression of *Cast_Gnk2*-like has been successfully achieved in both holm oak and cork oak through genetic transformation, leading to increased tolerance to Pc [20, 22]. In these species, transformed plants exhibited significantly improved survival rates and reduced root necrosis. Furthermore, overexpression of *GNK2-1* from *Ginkgo biloba* seed kernels in cucumber also demonstrated a stable and significant inhibitory effect on *Fusarium oxysporum* growth [64]. The antifungal activity of Gnk2 is attributed to the presence of the DUF26 domain, characteristic of cysteine-rich lectins and defense-related proteins. This domain confers lectin activity, allowing the protein to bind to mannose and mannan residues in fungal cell walls, thereby disrupting their integrity and inhibiting fungal growth [29].

It might be plausible that *Cast_Gnk2-like* gene contributes to the synergistic interaction between lectins and pectins within the plant root defense machinery against Pc. Lectins and pectin work together in a multi-layered defense system, where lectins act as sensors and pectin serves as a target or trigger for immune responses against pathogens in the root environment [65]. Recently, it has been demonstrated that Pc zoospores show pectinase activity during penetration of plant roots [46]. One might speculate that the effect of Cast_Gnk2-like might not only be a direct antimicrobial effect on Pc, but also improve the pectin barrier and lectin activity in roots, improving root protection against the penetration of zoospores. This hypothesis has been previously suggested for another antimicrobial proteins [66], but would require further molecular analysis for the case of Gnk2. Consequently, the overexpression of soluble lectins or lectin receptor kinases may reduce pathogen growth and may activate defense responses such as the production of reactive oxygen species (ROS), expression of pathogenesis-related genes, and the strengthening of cell walls, thereby enhancing the plant’s overall resistance to infections [67–69]. The mechanism seems very similar to that of glucanases or chitinases, which degrade key structural components of the pathogen’s cell wall. Taken together, these studies reinforce the idea that weakening the structural integrity of the pathogen’s cell wall, whether through molecular binding or enzymatic degradation, is an effective strategy to prevent infection. In turn, a broader theory is supported that a significant part of plant tolerance or resistance relies on the ability to recognize and target essential cell wall components of the pathogen, thereby blocking its colonization and progression. Overall, the studies concur that weakening the pathogen’s cell wall, whether by binding or enzymatic degradation, constitutes an effective means of preventing infection.

## Conclusions

The overexpression of the *Cast_Gnk2-like* gene in European chestnut significantly enhancedtolerance to *Phytophthora cinnamomi*. These findings support the use of antifungal protein overexpression as an effective strategy to improve resistance to fungal pathogens, and position *Cast_Gnk2-like* gene as a promising candidate for reinforcing oomycete resistance in genetically improved crops. The use of an endogenous gene from chestnut ensures better physiological compatibility, stable expression, and integration into native defense pathways. Furthermore, since *Cast_Gnk2-like* originates from the same genus, future development of cisgenic lines via genome editing, may be feasible, potentially circumventing some of the regulatory constrains associated with transgenic approaches. In addition to improved pathogen tolerance, genome editing offers the advantage of precise genetic modifications without the need for exogenous promoters or reporter genes, which can sometimes interfere with normal plant development. Finally, although the tolerance results are promising, it is necessary to confirm these data through ex vitro tolerance assays.

## Supplementary Information

Supplementary Material 1.

Supplementary Material 2.

## Data Availability

The protein sequence analyzed in this study (Cast_Gnk2-like) is publicly available in the NCBI Protein database and in the UniProt database under accession number P0DO63.
