# Supplementary material for: Overexpression of ginkbilobin-2 homologous domain gene to enhance the tolerance to Phytophthora cinnamomi in plants of European chestnut
Source: BMC Genomics. 2026 Jan 9;27:155. doi: 10.1186/s12864-025-12485-x (PMC12879325; doi:10.1186/s12864-025-12485-x)
Supplement: Supplementary file 1 — Supplementary Material 1. [file 12864_2025_12485_MOESM1_ESM.pdf]

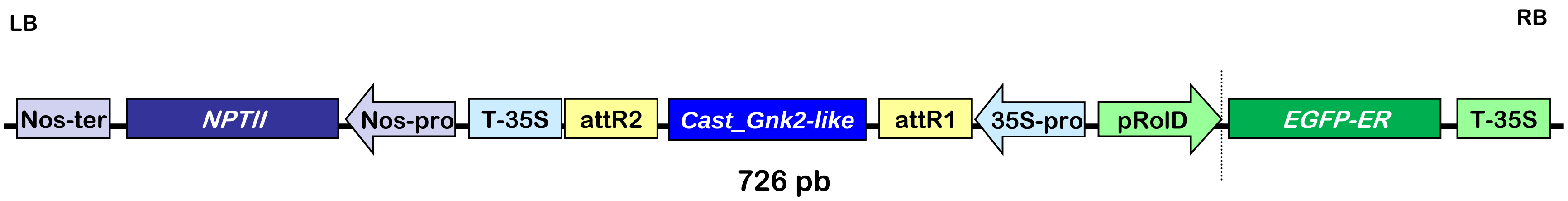

Supplementary information 1. A schematic representation of T-DNA region of the plasmid pK7WG2D-GIN. *nos-ter*, *nos-pro* terminator and promoter of nopaline synthase gene, respectively; *NPTII* neomycin phosphotransferase marker gene; *35S-pro, T-35S* promoter and terminator of Cauliflower mosaic virus gene, respectively; *EGFP-ER* green fluorescence protein gene; *Cast_Gnk2-like* gene encoding a Ginkbilobin-2 protein; *prolD* rol root loci D promoter; *RB* right border; *LB* left border.
