# Supplementary material for: Overexpression of ginkbilobin-2 homologous domain gene to enhance the tolerance to Phytophthora cinnamomi in plants of European chestnut
Source: BMC Genomics. 2026 Jan 9;27:155. doi: 10.1186/s12864-025-12485-x (PMC12879325; doi:10.1186/s12864-025-12485-x)
Supplement: Supplementary file 2 — Supplementary Material 2. [file 12864_2025_12485_MOESM2_ESM.pdf]

Supplementary information 2. Primers and Their Corresponding Amplification Conditions Used in the present Paper.

GENE	PRIMER NAME	PRIMER SEQUENCE (5′-3′)	PCR CONDITIONS	FRAGMENT AMPLIFIED (bp)	PURPOSE	Efficiency and R^2
NPTII	NPTII-F<br>NPTII-R	GTCATCTCACCTTGCTCCTGCC<br>AAGAAGGCGATAGAAGCGA	35 cycles:<br>94ºC x 30s<br>60ºC x 30s<br>72ºC x 42s	472	PCR analysis	-
GFP	EGFP-F<br>EGFP-R	CACCGGGGTGGTGCCCAT<br>CTAGTGGATCCCCCGGGC	40 cycles:<br>94ºC x 15s<br>56ºC x 30s<br>72ºC x 1min	740	PCR analysis	-
Cast_Gnk2-like	T35S-R<br>GIN-D	AGGTCACTGGATTTTGGT<br>CTGCCACTAGCCGTTATGGT	35 cycles:<br>98ºC x 10s<br>56ºC x 30s<br>72ºC x 1min	890	PCR analysis	-
NPTII	NPTII-F<br>NPTII-R	GGGCGCCCGGTTCTTTTTG<br>AGTCCCTCCCCGCTTCAGTG	Tm 60ºC	145	qPCR copy number	1.95<br>0.96
Cast_Gnk2-like	Cc_Gnk2-F<br>Cc_Gnk2-R	GGGGACCTAAAGCTTGACTCA<br>CATCGCAACAGTTGGGAAGTT	Tm 60ºC	129	qPCR transgene expression	1.80<br>0.94
Actin	Actin-F<br>Actin-R	CCTTGCTGGTCGTGATCTC<br>GTCTCAAGTTCCTGCTCATAGTC	Tm 62ºC	150	Reference gene for qPCR	1.80<br>0.99
Elongation factor 1 -α	EF1α-F<br>EF1α-R	CGGTTACTGAGTACTAGCCTTGC<br>CTGCCGAAGACCTTATTGAAAG	Tm 60ºC	84	Reference gene for qPCR	1.84<br>1.00

F: forward; R: reverse.
